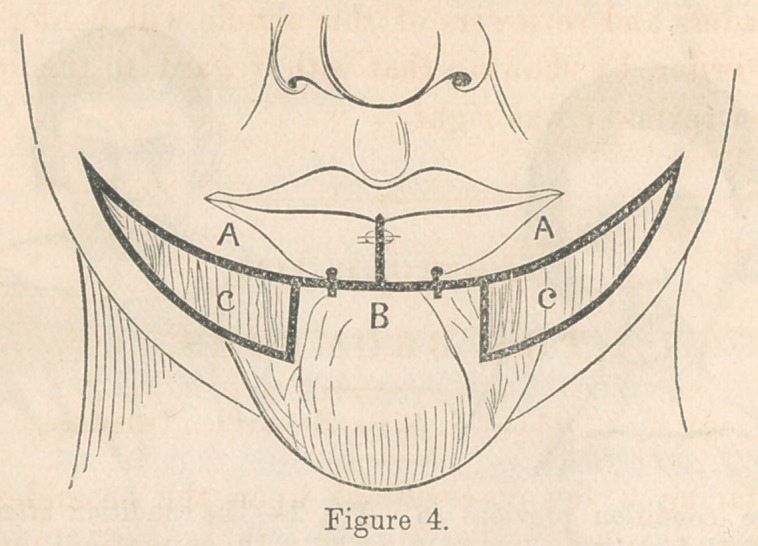# The Establishment of a Line of Immobility in Plastic Operations

**Published:** 1870-01

**Authors:** David Prince

**Affiliations:** Jacksonville, Ill.


ARTICLE II.

### THE ESTABLISHMENT OF A LINE OF IMMOBILITY IN PLASTIC OPERATIONS.

BY DAVID PRINCE, M.D., Jacksonville, Ill.

It is the purpose of this short article to ask the attention of the profession to an element in plastic surgery, first publicly suggested by the writer in a monograph on orthopedic surgery, published by Lindsay & Blackston, in 1866, and repeated at greater length, with an illustrative wood cut, in a Report on Plastics, made to the Illinois State Medical Society, in 1867, and also reprinted as a monograph.

The plan is so simple, that it would seem that it must have been thought of among the first expedients put in practice, but the writer has not been able to find any reference to it anywhere.

The plan is especially applicable in cases of depression and eversion of the lower lip, consequent upon the contraction of cicatrices from burns upon the neck and chest. Much ingenuity has been displayed in attempts to secure the replacing of the lip, so that the unfortunate sufferer might be able to hold his saliva, but the contraction of the cicatrical tissue upon the neck has in almost all cases drawn the lip down again.

The simple expedient adopted by the writer, is to make a curved incision across the neck, far enough below the chin, so that when the flesh is made to slide up in front of the chin, the lip shall be effectually bolstered up. The periosteum along the base of the jaw is scraped off so that the lower edge of the flap must attach itself to the bone. After this there can be no possibility of any traction upon the lip below.

Fig. 1.—The condition previous to the operation.

The lower lip is drawn down below the level of the shoulders.

Fig. 2.—The condition after the operation.

The lip is completely restored to its proper place and function, though the bridles upon the neck remain.

The accompanying wood cuts, Figures 1 and 2, illustrating a case of extreme eversion of the lip, and its complete restoration, may help to make the explanation more clear. The dotted line indicates the place of incision. The plan was not thought of until after other methods of sustaining the lip had failed.

Figure 3.

In order to show how great an advance this simple expedient is upon anything which had preceded it, the next two cuts, Figures 3 and 4 are introduced.

This is Teale's operation for eversion of the lower lip. A new lip is made upon the top of the old one.

Figure 4.

The heavy black line, A, B, C, shows the place and direction of the incision. The parts above this line are freely dissected up from the bone, and brought together, the vertical lines coming together at the middle line of the new lower lip. The corners of the mouth are extended by Dieffenbach's method, and the spaces C, C, are left to be covered by granulations.

A horrid deformity must always remain; while, by the plan introduced by the writer, no deformity whatever can be seen upon the face, and the bridles upon the neck are not made any worse by the restoration of the lip to its proper position.

The remaining deformities of the neck and chest can be treated by subsequent transplantations of integument. If this treatment for the neck and chest should not be attempted, or if it should be unsuccessful, the most important object, the restoration of the lip will have been accomplished.

This communication might seem unnecessary, and it would be, except for the fact that proper credit has been withheld by high authority. A Boston reviewer of the Report on Plastics, in which the operation was first carefully described, said of the report, in substance, that the portions which were claimed as new were not new, and what were admitted to be old had been better written in books everywhere accessible.

The operation is understood to have been very successfully repeated in Chicago, in a public institution, without any notice of its origin.

The readers and reviewers of this article will confer a favor upon the writer, by showing that with regard to this operation the Boston reviewer was right.

## Figures and Tables

**Fig. 1. f1:**
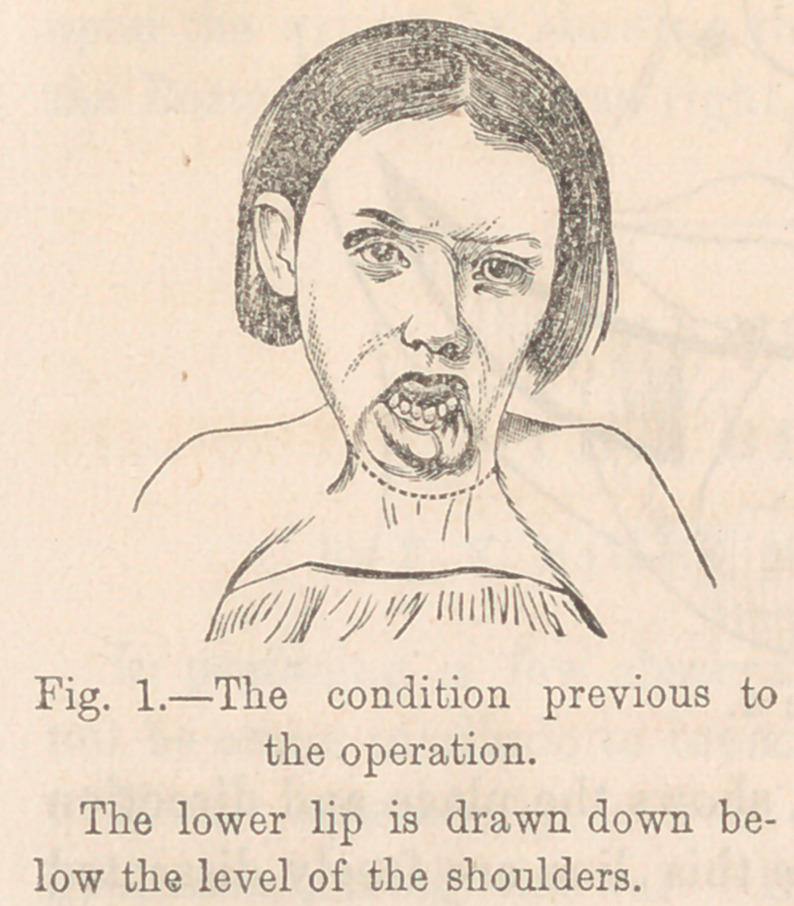


**Fig. 2. f2:**
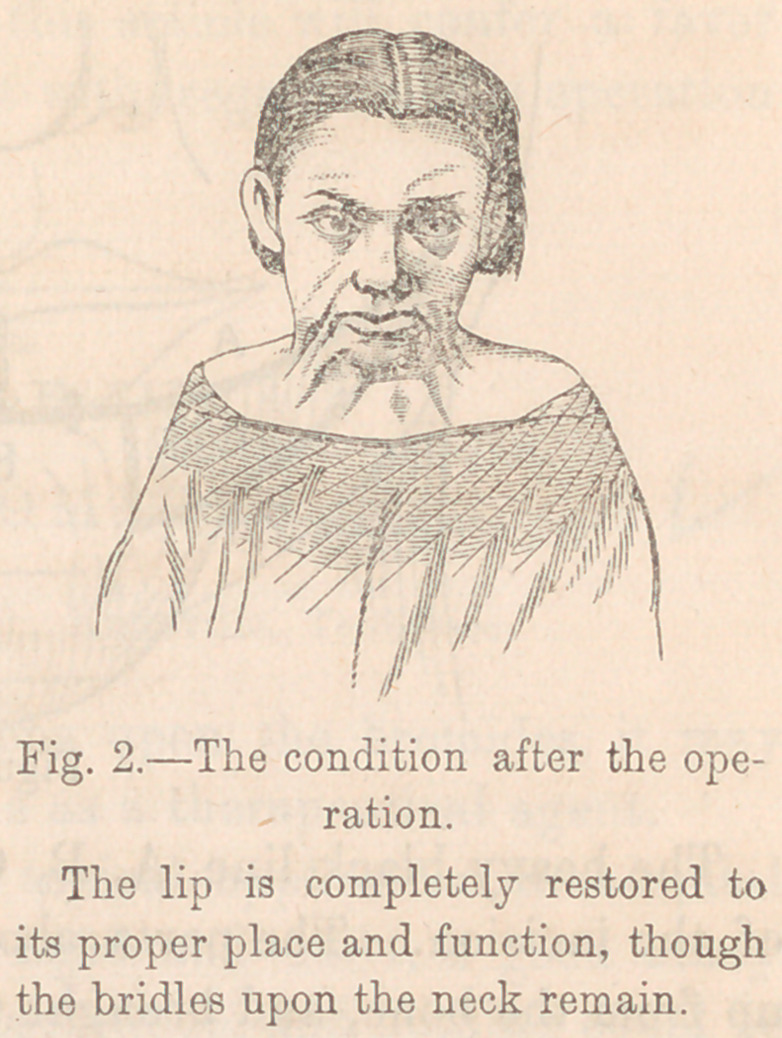


**Figure 3. f3:**
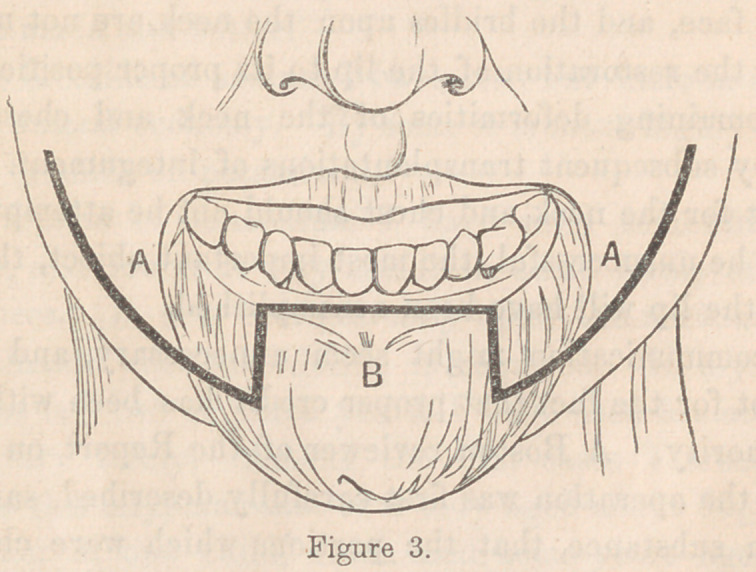


**Figure 4. f4:**